# Carbohydrate antigen 125 and risk of heart failure readmissions in patients with heart failure and preserved ejection fraction

**DOI:** 10.1038/s41598-022-05328-2

**Published:** 2022-01-25

**Authors:** Gema Miñana, Rafael de la Espriella, Patricia Palau, Pau Llácer, Eduardo Núñez, Enrique Santas, Ernesto Valero, Miguel Lorenzo, Gonzalo Núñez, Vicente Bodí, Raquel Heredia, Juan Sanchis, Antoni Bayés-Genís, Francisco J. Chorro, Julio Núñez

**Affiliations:** 1 Cardiology Department, Hospital Clínico Universitario de Valencia, Universitat de Valencia, INCLIVA, Avda. Blasco Ibáñez 17, 46010 Valencia, Spain; 2 CIBER Cardiovascular, Madrid, Spain; 3 Internal Medicine Department. Hospital, Universitario Ramón y Cajal, Madrid, Spain; 4 Cardiology Department and Heart Failure Unit, Hospital Universitari Germans Trias i Pujol, Badalona, Spain; 5 Department of Medicine, Autonomous University of Barcelona, Barcelona, Spain

**Keywords:** Biomarkers, Cardiology

## Abstract

We aimed to assess the association between CA125 and the long-term risk of total acute heart failure (AHF) admissions in patients with an index hospitalization with AHF and preserved ejection fraction (HFpEF). We prospectively included 2369 patients between 2008 and 2019 in three centers. CA125 and NT-proBNP were measured during early hospitalization and evaluated as continuous and categorized in quartiles (Q). Negative binomial regressions were used to assess the association with the risk of recurrent AHF admission. The mean age of the sample patients was 76.7 ± 9.5 years and 1443 (60.9%) were women. Median values of CA125 and NT-proBNP were 38.3 (19.0–90.0) U/mL, and 2924 (1590–5447) pg/mL, respectively. During a median follow-up of 2.2 (0.8–4.6) years, 1200 (50.6%) patients died, and 2084 AHF admissions occurred in 1029 (43.4%) patients. After a multivariate adjustment, CA125, but not NT-proBNP, was positively and non-linearly associated with the risk of cumulative AHF-readmission (*p* < 0.001). Compared to Q1, patients belonging to Q2, Q3, and Q4 showed a stepwise risk increase (IRR = 1.29, 95% CI 1.08–1.55, *p* = 0.006; IRR = 1.35, 95% CI 1.12–1.63, *p* = 0.002; and IRR = 1.62, 95% CI 01.34–1.96, *p* < 0.001, respectively). In conclusion, CA125 predicted the risk of long-term AHF-readmission burden in patients with HFpEF and a recent admission for AHF.

## Introduction

Among patients with acute heart failure (AHF), those with heart failure and preserved ejection fraction (HFpEF) account for 40% to 55%^1^. Patients with HFpEF display a heterogeneous clinical and pathophysiological profile^2,3^. However, the clinical presentation and the risk of mortality and readmission are similar to those reported for heart failure and reduced ejection fraction (HFrEF)^3,4^.

Carbohydrate antigen 125 (CA125) is a large mucin synthesized by mesothelial cells. Higher values of this glycoprotein have been reported in patients with several conditions, including AHF syndromes^5^. During the last 2 decades, this biomarker consolidated its role as a surrogate of fluid overload and right heart failure (HF) phenotype in this acute scenario^5–8^. CA125 has also been independently associated with a higher risk of time to first adverse clinical outcomes in patients with AHF, mainly within the first year of follow-up^7,8^. Predicting the risk of recurrent/total hospitalizations, especially in HFpEF, remains an unmet clinical need^9^.

This study aimed to assess the association between CA125 and the risk of long-term recurrent HF-readmission in patients with HFpEF recently discharged for AHF. Additionally, we evaluated the association of amino-terminal pro-brain natriuretic peptide (NT-proBNP) with the same endpoint.

## Methods

### Study population

We retrospectively studied a cohort of 4812 patients consecutively discharged after admission for AHF at three tertiary-care hospitals in Valencia, Spain, from January 1st, 2008, to October 1st, 2019. AHF was diagnosed according to the definition proposed by guidelines^10,11^. By design, our analysis excluded patients with left ventricle ejection fraction (LVEF) < 50% (n = 2204), and those who underwent valve surgical replacement or transcatheter valvular intervention (n = 128), or died during hospital stay (n = 111). The final study sample included 2369 patients (Supplementary Fig. 1).

Pre-established electronic questionnaires were used during admission to record information related to demography, medical history, vital signs, physical examination, 12-lead electrocardiogram, echocardiogram, and medical treatment on discharge. The study protocol conformed to the ethical guidelines of the 1975 Declaration of Helsinki (revised in 1983), as reflected by an a priori approval by the institution's human research committee. and the local ethics committee (Comité Ético de Investigación del Hospital Clínico Universitario de Valencia) approved the study. All participants provided written informed consent. Patients were not involved in the design and conduct of this research.

### Biomarker assessment

Plasma CA125 and NT-proBNP were measured together within the first 24–48 h after admission and analyzed in the local laboratory at each center using commercially available immunoassays (Elecsys® NT-proBNP assay, Roche Diagnostics; Elecsys® CA125 II assay, Roche Diagnostics). For CA125, the intra-assay precision (coefficient of variation) is 1.4%–2.0%, and the inter-assay precision (coefficient of variation) is 0.0%–0.9%, with an analytical range of 0.6–5000 U/mL^12^. For NT-proBNP, the intra-assay precision (coefficient of variation) is 1.2%–1.5%, and the inter-assay precision (coefficient of variation) is 4.4%–5.0%, with an analytical range of 5–35,000 pg/mL^13^.

### Echocardiographic evaluation

After clinical stabilization, a comprehensive transthoracic echocardiographic examination was performed using commercially available systems (Agilent Sonos 5500 or IE33 Philips, MA, USA). Two-dimensional and Doppler measurements were performed and analyzed by trained cardiologists using standard views and techniques. Preserved LVEF was defined as ≥ 50% in the transthoracic echocardiographic examination performed during admission^10^.

### Follow-up and outcomes

Total HF readmissions occurring during the follow-up were selected as the endpoint of interest. Additionally, we evaluated all-cause mortality and cardiovascular mortality as endpoints. We identified HF-admission and fatal events from the electronic clinical records of the Regional Health Care System. The personnel in charge of endpoint adjudication were not aware of the patient's levels of both biomarkers.

### Statistical analysis

Continuous variables are expressed as mean ± standard deviation (SD) or median [interquartile interval (IQI], as appropiate. Categorical variables are presented as percentages. Baseline characteristics among CA125 and NT-proBNP quartiles were compared by ANOVA, Kruskal–Wallis, or chi-squared tests, as appropriate. Rates of events were presented as per 100 person-years (P-Y). To account for the positive correlation between HF-hospitalization and mortality, we fitted the Famoye bivariate Poisson regression model. The number of admissions (as counts) and mortality (as the terminal event) were modeled simultaneously and linked by shared frailty. To account for differences in the time to each recurrent event, the log of follow-up time was included as an offset in each submodel. Crude and adjusted rates (number of events per 100 P-Y) are presented among the groups tested. We selected explanatory variables for the initial multivariate model based on subject-matter knowledge. Then, using a backward elimination procedure that included a polynomial transformation for continuous variables, we obtained a final model. In some instances, however, the automatic selection procedure was overridden by leaving well-known predictors in the setting of HF regardless the *p*-value, as priorly described in previous works^14^.

The final covariates included in the recurrent HF-readmission, all-cause mortality, and cardiovascular mortality model were: age, sex, hypertension, first admission for AHF, last New York Heart Association (NYHA) class III vs I-II under stable condition, ischemic and heart valve disease, Charlson comorbidity index, atrial fibrillation, heart rate, and their interaction (atrial fibrillation*heart rate), hemoglobin, estimated glomerular filtration rate (eGFR), left atrial diameter, tricuspid annular plane systolic excursion (TAPSE), furosemide equivalent dose at discharge, and treatment at discharge with angiotensin-converting enzyme inhibitors (ACEI) or angiotensin receptor blockers (ARB), aldosterone antagonists, and beta-blockers.

Risk estimates are presented as incidence rate ratios (IRRs). We set a two-sided *p*-value < 0.05 as the threshold for significance. All analyses were performed in Stata 15.1 (Stata Statistical Software, Release 15 [2017]; StataCorp LP, College Station, TX, USA). We used the "Bivcnto" Stata module for multivariate and bivariate Poisson analyses.

## Results

### Baseline characteristics

The mean age was 76.7 ± 9.5 years, 1443 (60.9%) patients were women, and 880 (37.1%) had a prior diagnosis of HF. Most of the patients had a prior history of hypertension (83.4%) and were admitted for the first time (68.4%). Median (IQR) values of CA125 and NT-proBNP were 38.3 (19.0–90.0) U/mL, and 2924 (1590–5447) pg/mL, respectively. The baseline characteristics across CA125 and NT-proBNP quartiles are presented in Tables 1 and 2.

**Table 1:** Baseline characteristics across CA125 quartiles.

Variables	CA125 Q1 (N = 592)	CA125 Q2 (N = 592)	CA125 Q3 (N = 593)	CA125 Q4 (N = 592)	*p*-value
**Demographics and medical history**					
Age, years	76.9 ± 9.3	77.1 ± 9.2	76.9 ± 9.3	75.7 ± 10.3	0.071
Gender (male), n (%)	223 (37.7)	235 (39.7)	220 (37.1)	248 (41.9)	0.317
Hypertension, n (%)	522 (88.2)	507 (85.6)	484 (81.6)	463 (78.2)	< 0.001
Diabetes Mellitus, n (%)	233 (39.4)	266 (44.9)	244 (41.1)	247 (41.7)	0.268
Dyslipidemia, n (%)	306 (51.7)	321 (54.2)	299 (50.4)	280 (47.3)	0.117
Smoker, n (%)	36 (6.1)	40 (6.8)	37 (6.2)	53 (8.9)	0.186
Prior smoker, n (%)	123 (20.8)	130 (22.0)	117 (19.7)	135 (22.8)	0.592
IHD, n (%)	164 (27.7)	160 (27.0)	147 (24.8)	131 (22.1)	0.114
Valve heart disease, n (%)	183 (30.9)	202 (34.1)	227 (38.3)	253 (42.7)	< 0.001
Prior history of HF, n (%)	214 (36.1)	221 (37.3)	235 (39.6)	210 (35.5)	0.469
Prior AHF admission, n (%)	196 (33.1)	201 (33.9)	190 (32.0)	161 (27.2)	0.057
Charlson index, points	2 (1–3)	2 (1–3)	2 (1–3)	2 (1–3)	0.563
Pleural effusion, n (%)	150 (25.3)	243 (41.0)	322 (54.3)	416 (70.3)	< 0.001
Peripheral edema, n (%)	310 (52.4)	363 (61.3)	402 (67.8)	439 (74.2)	< 0.001
NYHA III-IV prior to admission, %	94 (15.9)	92 (15.5)	107 (18.0)	118 (19.9)	0.158
**Vital signs**					
Heart rate, bpm	92 ± 28	93 ± 29	95 ± 29	96 ± 30	0.487
SBP, mmHg	150 ± 32	147 ± 31	150 ± 31	143 ± 30	0.200
DBP, mmHg	80 ± 19	78 ± 19	78 ± 18	77 ± 17	0.058
**Electrocardiogram**					
Atrial fibrillation, n (%)	288 (48.6)	289 (48.8)	336 (56.7)	372 (62.8)	< 0.001
BBB, n (%)	155 (26.2)	144 (24.3)	132 (22.3)	123 (20.8)	0.136
**Echocardiography**					
LVEF, %	62.2 ± 7.1	62.0 ± 7.1	61.8 ± 7.4	61.2 ± 7.5	0.317
LAD, mm	43.2 ± 7.5	43.5 ± 7.6	44.2 ± 7.4	45.1 ± 7.7	0.784
TAPSE, mm	20.4 ± 3.0	19.8 ± 3.5	19.4 ± 3.6	18.6 ± 3.2	< 0.001
**Laboratory data**					
Hemoglobin, g/dL	12.5 ± 1.9	12.1 ± 1.9	11.8 ± 1.8	12.0 ± 1.9	0.625
eGFR (MDRD formula), mL/min/1.73m^2^	63.8 ± 25.9	62.3 ± 32.0	60.3 ± 26.6	63.0 ± 33.4	< 0.001
Serum sodium, mEq/L	139 ± 4	138 ± 4	138 ± 5	138 ± 5	< 0.001
Serum potassium, mEq/L	4.2 ± 0.5	4.3 ± 0.5	4.3 ± 0.5	4.3 ± 0.5	0.022
NT-proBNP, pg/mL*	2210 (1170–3726)	2682 (1561–4998)	3397 (1932–6200)	3454 (1910–6368)	< 0.001
CA125, U/mL*	12.0 (8.3–15.5)	26.8 (22.5–32.0)	57.1 (47.0–72.0)	153.1 (115–227.4)	< 0.001
**Medical treatment at discharge**					
Beta-blockers, n (%)	368 (62.2)	378 (63.8)	409 (69.0)	384 (64.9)	0.088
ACEI or ARB or ARNI, n (%)	287 (63.2)	278 (61.2)	274 (57.2)	310 (57.9)	0.195
MRA, n (%)	52 (17.7)	38 (14.7)	43 (15.7)	69 (22.9)	0.048
Oral anticoagulation, n (%)	285 (48.4)	296 (50.3)	299 (51.2)	353 (60.1)	< 0.001
**Outcomes**					
Death, rates per 100 P-Y	12.1	15.0	17.7	19.5	< 0.001
Total HF-readmissions, rates per 100 P-Y	30.7	42.7	41.3	46.2	0.004

AHF: acute heart failure; BBB: bundle branch block; CA125: carbohydrate antigen 125; DBP: diastolic blood pressure; eGFR: estimated glomerular filtration rate; ID: iron deficiency; IHD: ischemic heart disease; LAD: left atrial diameter; MDRD: Modification of Diet in Renal Disease; MRA: mineralocorticoid receptor antagonists; NT-proBNP: amino-terminal pro-brain natriuretic peptide; NYHA: New York Heart Association; PASP: pulmonary artery systolic pressure; P-Y: person-years; SBP: systolic blood pressure; TAPSE: tricuspid annular plane systolic excursion; TSAT: transferrin saturation; WHO: World Heart Organization.

Values for continuous variables are expressed as mean ± standard deviation.

*Values expressed as mean (interquartile range).

CA125 quartiles: Q1 = 1.4–19 U/mL; Q2 = 19–38.26 U/mL; Q3 = 38.3–90 U/mL; Q4 = 90–1500 U/mL.

**Table 2:** Baseline characteristics across NT-proBNP quartiles.

Variables	Q1 (N = 592)	Q2 (N = 592)	Q3 (n = 593)	Q4 (n = 592)	*p*-value
**Demographics and medical history**					
Age, years	72.6 ± 10.5	75.7 ± 8.6	78.0 ± 9.2	80.4 ± 8.0	< 0.001
Gender (male), n (%)	246 (41.5)	229 (38.7)	232 (39.1)	219 (37.0)	0.450
Hypertension, n (%)	491 (82.9)	486 (82.1)	491 (82.8)	508 (85.8)	0.326
Diabetes Mellitus, n (%)	266 (44.9)	261 (44.1)	244 (41.5)	219 (37.0)	0.025
Dyslipidemia, n (%)	312 (52.7)	286 (48.3)	291 (49.1)	317 (53.5)	0.186
Smoker, n (%)	64 (10.8)	30 (5.1)	44 (7.4)	28 (4.7)	< 0.001
Prior smoker, n (%)	121 (20.4)	141 (23.9)	121 (20.4)	122 (20.6)	0.395
IHD, n (%)	134 (22.6)	145 (24.5)	165 (27.8)	158 (26.7)	0.172
Valve heart disease, n (%)	202 (34.1)	181 (30.6)	223 (37.6)	259 (43.7)	< 0.001
Prior history of HF, n (%)	178 (30.1)	194 (32.8)	248 (41.8)	260 (43.9)	< 0.001
Prior AHF admission, n (%)	148 (25.0)	194 (32.8)	209 (35.2)	197 (33.3)	0.001
Charlson index, points	1 (1–3)	1 (1–3)	2 (1–3)	2 (1–4)	< 0.001
Pleural effusion, n (%)	207 (35.0)	284 (48.0)	306 (51.6)	334 (56.4)	< 0.001
Peripheral edema, n (%)	330 (55.7)	382 (64.5)	401 (67.6)	401 (67.7)	< 0.001
NYHA III-IV prior to admission, %	92 (15.5)	81 (13.7)	99 (16.7)	139 (23.5)	< 0.001
**Vital signs**					
Heart rate, bpm	92 ± 29	93 ± 30	98 ± 30	94 ± 28	0.414
SBP, mmHg	153 ± 33	150 ± 31	145 ± 29	140 ± 29	0.004
DBP, mmHg	82 ± 20	79 ± 18	78 ± 18	75 ± 17	0.014
**Electrocardiogram**					
Atrial fibrillation, n (%)	271 (45.8)	333 (56.2)	350 (59.0)	331 (22.9)	< 0.001
BBB, n (%)	117 (19.8)	130 (22.0)	147 (24.8)	160 (27.0)	0.018
**Echocardiography**					
LVEF, %	63.4 ± 7.3	62.2 ± 7.2	61.3 ± 6.8	60.3 ± 7.5	0.095
LAD, mm	43.5 ± 7.6	43.7 ± 7.7	44.1 ± 7.5	44.6 ± 7.4	0.717
TAPSE, mm	20.4 ± 3.5	19.7 ± 3.1	19.2 ± 3.2	18.9 ± 3.6	< 0.001
**Laboratory data**					
Hemoglobin, g/dL	12.7 ± 2.09	12.2 ± 1.9	12.0 ± 1.8	11.5 ± 1.8	0.147
eGFR (MDRD formula), mL/min/1.73m^2^	75.1 ± 33.9	66.6 ± 29.6	59.4 ± 23.8	48.2 ± 23.3	< 0.001
Serum sodium, mEq/L	139 ± 4	139 ± 4	139 ± 4	138 ± 5	< 0.001
Serum potassium, mEq/L	4.2 ± 0.5	4.2 ± 0.5	4.3 ± 0.5	4.4 ± 0.6	< 0.001
NT-proBNP, pg/mL*	1014 (677–1306)	2200 (1869–2500)	3848 (3374–4431)	8658 (6603–13,367)	< 0.001
CA125, U/mL*	25.8 (14–65.4)	36.0 (19.15–84.3)	39.4 (19.2–92.0)	54.2 (27.0–110.2)	< 0.001
**Medical treatment at discharge**					
Beta blockers, n (%)	366 (61.8)	387 (65.4)	390 (65.7)	396 (66.9)	0.291
ACEI or ARB, n (%)	352 (69.2)	302 (63.4)	267 (57.2)	228 (48.5)	< 0.001
MRA, n (%)	63 (21.6)	33 (14.0)	51 (19.0)	55 (16.6)	0.121
Oral anticoagulation, n (%)	282 (47.9)	321 (54.4)	323 (55.4)	307 (52.4)	0.049
**Outcomes**					
Death, rates per 100 P-Y	9.5	14.2	20.1	27.4	< 0.001
Total HF-readmissions, rates per 100 P-Y	29.4	36.0	45.3	55.0	< 0.001

ACEI: angiotensin converting enzyme inhibitors; AHF: acute heart failure; ARB: angiotensin receptor blockers; BBB: bundle branch block; CA125: carbohydrate antigen 125; DBP: diastolic blood pressure; eGFR: estimated glomerular filtration rate; ID: iron deficiency; IHD: ischemic heart disease; LAD: left atrial diameter; MDRD: Modification of Diet in Renal Disease; MRA: mineralocorticoid receptor antagonists; NT-proBNP: amino-terminal pro-brain natriuretic peptide; NYHA: New York Heart Association; PASP: pulmonary artery systolic pressure; P-Y: person-years; SBP: systolic blood pressure; TAPSE: tricuspid annular plane systolic excursion; TSAT: transferrin saturation; WHO: World Heart Organization.

Values for continuous variables are expressed as mean ± standard deviation.

*Values expressed as mean (interquartile range).

NT-proBNP quartiles: Q1 = 24–1589 pg/mL; Q2 = 1590–2922 pg/mL; Q3 = 2924–5447 pg/mL; Q4 = 5450–35,000 pg/mL.

Patients in the upper quartiles of CA125 displayed more conditions of valvular etiology and more features of congestion. They also showed higher atrial fibrillation rates, lower sodium and TAPSE, and higher NT-proBNP (Table 1).

Those patients in the upper quartiles of NT-proBNP exhibited a worse baseline risk profile. They were older, had a higher incidence of heart valve disease, prior hospitalizations, more parameters of congestion (including higher CA125 values), and a higher proportion of bundle branch block. In addition, they showed lower systolic and diastolic blood pressure, TAPSE, hematocrit, and eGFR. They were discharged receiving less frequently ACEI or ARB (Table 2).

### Recurrent AHF-admission risk

At a median follow-up of 2.2 years (0.8–4.6), 1029 (43.4%) patients had at least 1 HF-admission. During the follow-up, the episodes of total HF-readmission were 2084. Most patients who were readmitted showed a single HF admission (56.6%). The percentage of patients with 2, 3, 4, and > 4 HF admissions were 22.6%, 10.0%, 5.0%, and 5.7%, respectively.

Unadjusted rates for recurrent HF admissions were higher when moving from low to high CA125 and NT-proBNP quartiles. For CA125, the readmission rates were 30.7, 42.7, 41.3, and 46.2 per 100 P-Y for Q1 to Q4, respectively (*p* = 0.004). The rates for NTproBNP were 29.4, 36.0, 45.3, and 55.0 per 100 P-Y for Q1 to Q4, respectively (*p* < 0.001).

**Figure 1:**
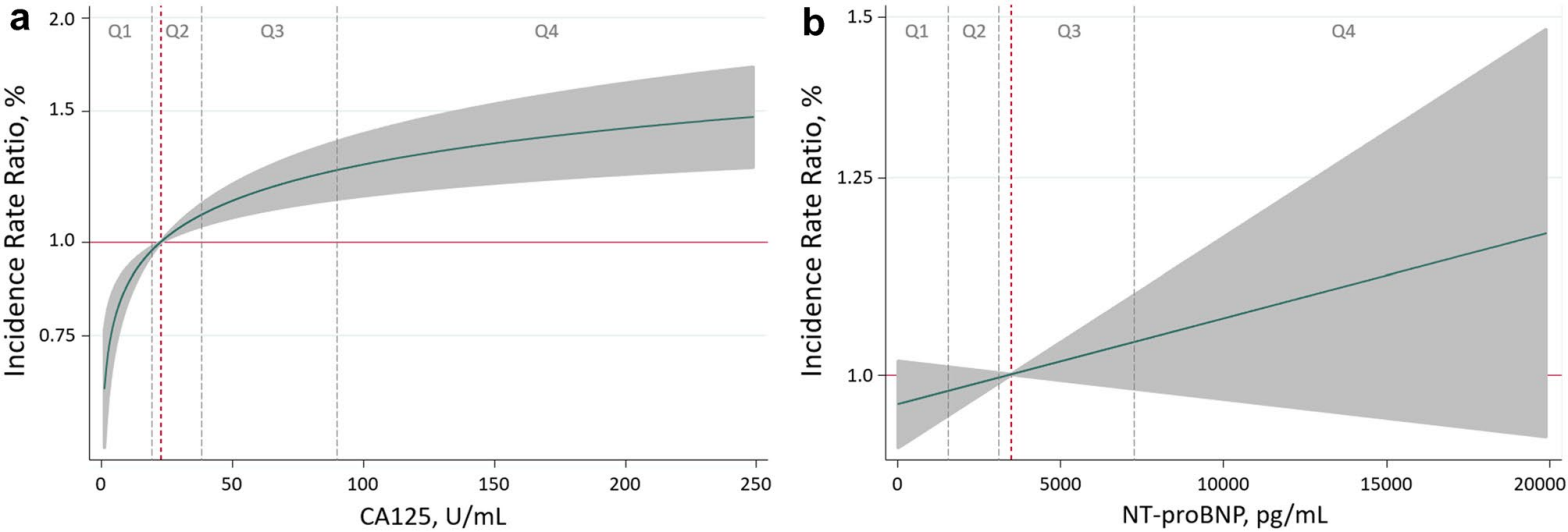
Functional form of the risk of AHF recurrent hospitalizations among the continuum of CA125 and NT-proBNP values. (**a**) CA125. (**b**) NT-proBNP. AHF: acute heart failure; CA125: carbohydrate antigen 125; NT-proBNP: amino-terminal pro-brain natriuretic peptide. CA125 quartiles: Q1 = 1.4–19 U/mL; Q2 = 19–38.26 U/mL; Q3 = 38.3–90 U/mL; Q4 = 90–1500 U/mL.

**Figure 2:**
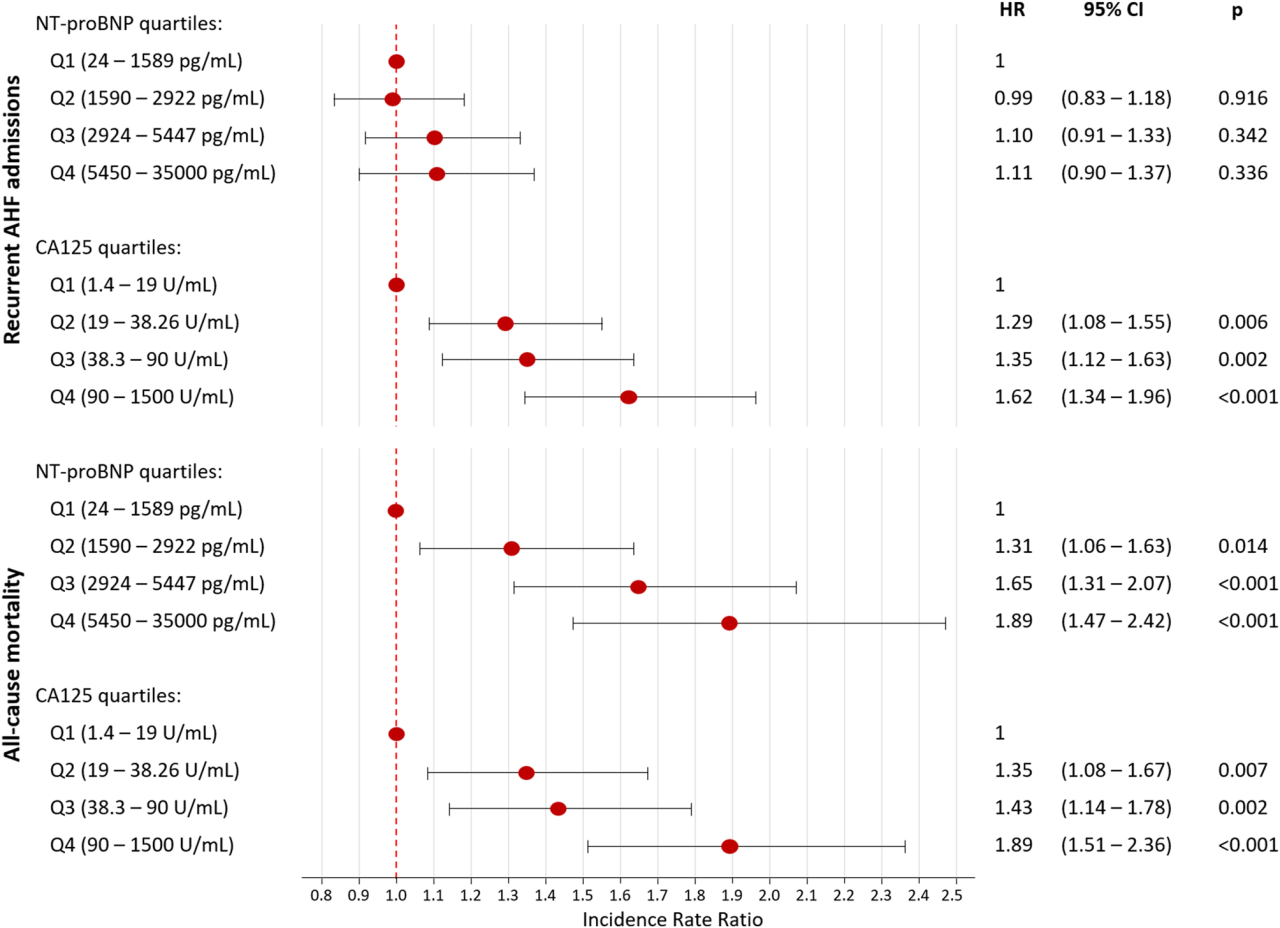
Risk of AHF recurrent hospitalizations and all-cause mortality among NT-proBNP and CA125 quartiles. AHF: acute heart failure; CA125: carbohydrate antigen 125; NT-proBNP: amino-terminal pro-brain natriuretic peptide.

After multivariate adjustment, CA125 remained positively associated with the HF-readmission risk. CA125 was non-linearly associated with higher risk (*p* < 0.001), as shown in Fig. 1a. A pronounced increase in risk was observed within theoretically normal values (< 35 U/mL). Above this value, the increase in risk was also progressive but of lesser magnitude (Fig. 1a). When categorized in quartiles, and compared to Q1 (< 19 U/ml), those in the Q2 (19–38.26 U/mL), Q3 (38.3–90 U/mL), and Q4 (> 90 U/mL), showed a stepwise risk increase in total AHF readmissions (Q2 vs Q1: IRR = 1.29, 95% CI 1.08–1.55, *p* = 0.006; Q3 vs Q1: IRR = 1.35, 95% CI 1.12–1.63, *p* = 0.002; and Q4 vs. Q1: IRR = 1.62, 95% CI 1.34–1.96, *p* < 0.001, respectively) as also shown in Fig. 2.

Contrary to CA125, after multivariate analyses, NT-proBNP was no longer associated with the burden of total HF readmissions when evaluated along the continuum (Fig. 1b) or categorized in quartiles (Fig. 2).

### Risk of AHF-readmission: subgroup analyses

With the exception of diabetes (*p*-value for interaction = 0.005), there was no evidence for a differential predictive value of CA125 among the most relevant subgroups. Thus, CA125 along its continuum was homogeneously associated with higher readmission risk across age, sex, renal function, comorbidity burden, atrial fibrillation, and ischemic heart disease. In patients with diabetes, CA125 portrends a greater risk (Supplementary Figs. 2 and 3).

### Mortality risk

**Figure 3:**
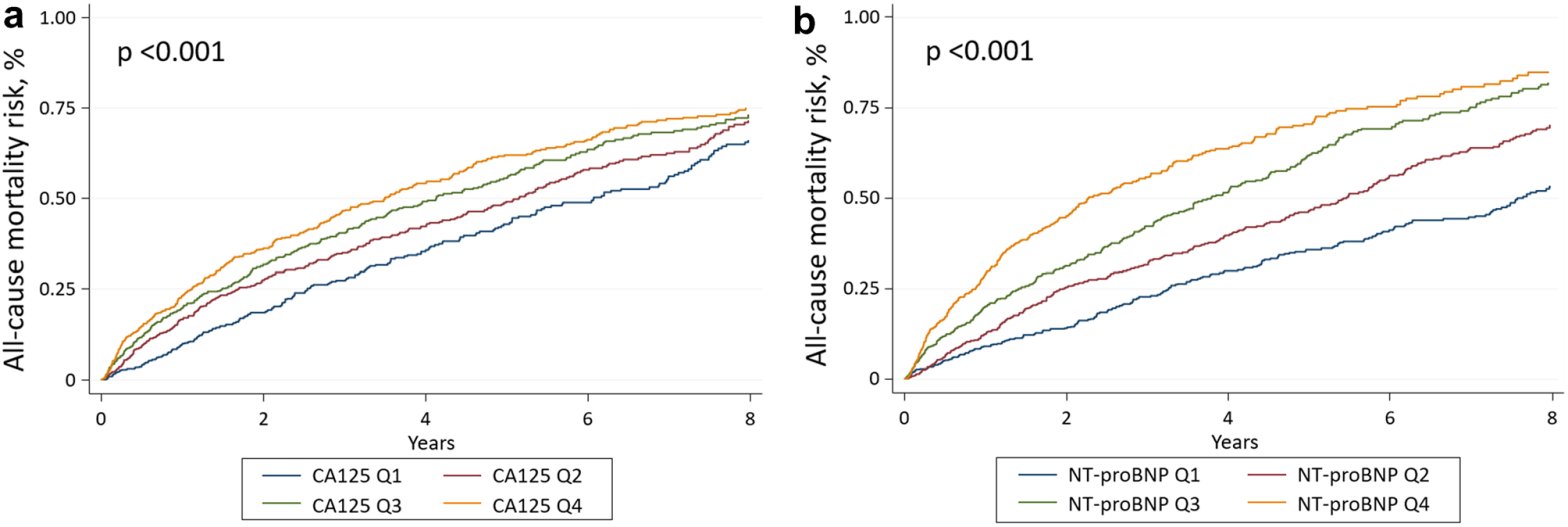
All-cause mortality risk across CA125 and NT-proBNP quartiles. (**a**) CA125 quartiles. (**b**) NT-proBNP quartiles. CA125: carbohydrate antigen 125; NT-proBNP: amino-terminal pro-brain natriuretic peptide. CA125 quartiles: Q1 = 1.4–19 U/mL; Q2 = 19–38.26 U/mL; Q3 = 38.3–90 U/mL; Q4 = 90–1500 U/mL. NT-proBNP quartiles: Q1 = 24–1589 pg/mL; Q2 = 1590–2922 pg/mL; Q3 = 2924–5447 pg/mL; Q4 = 5450–35,000 pg/mL.

A total of 1200 (50.7%) patients died during the follow-up, 825 of them were cardiovascular deaths (34.8% of the patients). The crude incidence rates for all-cause mortality among CA125 and NT-proBNP quartiles were higher when moving from lower to higher quartiles (Tables 1 and 2). Kaplan Meier plots showed divergent trajectories among quartiles of both biomarkers throughout the follow-up (Fig. 3).

**Figure 4:**
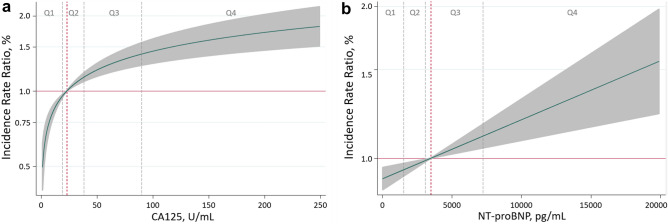
Functional form of the risk of all-cause mortality among the continuum of CA125 and NT-proBNP values. (**a**) CA125. (**b**) NT-proBNP. CA125: carbohydrate antigen 125; NT-proBNP: amino-terminal pro-brain natriuretic peptide. CA125 quartiles: Q1 = 1.4–19 U/mL; Q2 = 19–38.26 U/mL; Q3 = 38.3–90 U/mL; Q4 = 90–1500 U/mL. NT-proBNP quartiles: Q1 = 24–1589 pg/mL; Q2 = 1590–2922 pg/mL; Q3 = 2924–5447 pg/mL; Q4 = 5450–35,000 pg/mL.

Under the same multivariate setting and accounting for HF-readmission burden, CA125 remained associated with a higher risk of all-cause mortality (Fig. 4). This relationship was positive and non-linear, as shown in Fig. 4. Compared to Q1, those in Q2, Q3, and Q4 exhibited an stepwise risk increase (Fig. 2). Likewise, NT-proBNP was independently and linearly associated with a higher risk of this endpoint (Fig. 4). Estimates of risk for quartiles of NTproBNP are presented in Fig. 2. Similar results were found for CA125 and NTproBNP when cardiovascular death was analysed (Supplementary Fig. 4).

## Discussion

In this study, which included a large cohort of patients with HFpEF discharged after an episode of AHF, CA125, and not NT-proBNP predicted the long-term burden of total HF admissions. The predictive value of CA125 regarding HF-readmission was endorsed by consistent findings in the most representative clinical subgroups. Both biomarkers were associated with the risk of long-term all-cause mortality.

Hospitalizations due to HF decompensation are the leading cause of admission in patients older than 65 years in Western countries^15^. They account for the more significant part of HF-related morbidity and health care expenditure of the HF syndrome^2,4^. Prior studies assessing the factors associated with a higher risk of time to the first readmission have failed to find well-recognized prognostic factors. Additionally, most of the models have revealed a low discriminative ability for predicting readmissions^16^. Several reasons might explain these discouraging findings. Among them, the lack of homogeneous criteria for diagnosis and admission seems relevant. The variability in healthcare resources has also been postulated as crucial^16,17^.

Additionally, this "time-to-first" event methodology has been extensively criticized because it ignores all the subsequent outcomes occurring after the first event. Thus, given that a substantial number of patients experience recurrent admissions, this approach does not seem an accurate metric for measuring the morbidity burden of HF^9^. More recently, several authors have argued in favor of replacing analyses of time-to-first readmission by other statistical approaches that include the total number of admissions occurring during the course of the disease. Thus, recent studies and clinical trials have included total admissions during the study follow-up as objectives^18–20^. For instance, in the PARAGON trial, that evaluated the effect of sacubitril-valsartan vs. valsartan in 4822 patients with HFpEF, the primary endpoint was a composite of total HF-admission and death from cardiovascular causes^19^. This repeated events methodology may increase the ability to detect treatment effects to a greater extent than the classic "time-to-first" event methodology^9^. For instance, a post-hoc reanalysis of the CHARM-Preserved trial showed a superiority of candesartan when recurrent events were analyzed^21^.

HFpEF constitutes a syndrome with a wide variability of phenotypes and heterogeneous clinical course^2,22^. The risk of HF-admission is significantly higher in the first months following a decompensation^4,10^ regardless of LVEF. In a recent study of our group, including 2013 patients with AHF admission, we found that almost 70% of the patients were readmitted in the follow-up at least once, with up to nearly 30% of them having three or more readmissions^4^. We found that the total readmission burden was similar in HFpEF and HFrEF, while readmissions for non-cardiovascular causes were more freqüent in patients with HFpEF^4^.

CA125 has emerged as a valuable biomarker of congestion in AHF^5^. Although the pathophysiological mechanisms responsible for the increase of the synthesis of CA125 in AHF are not fully understood, mesothelial cells activation in response to increased hydrostatic pressure, mechanical stress and/or cytokine activation have been suggested as the crucial mechanisms^5,23,24^. Recently, our group reported that the most important factors related to CA125 in patients with AHF were, in order of importance, the presence of pleural effusion and the severity of tricuspid regurgitation^25^. Thus, we envision CA125 levels as a proxy of fluid overload and right-sided HF^25^. Thus, given that presence of fluid overload is highly prevalent in decompensated HF patients, it seems feasible to speculate that higher CA125 identified more advanced patients with greater congestion and a higher risk of new HF decompensations.

Conversely, natriuretic peptides are the standard HF biomarker accurately reflecting the high filling ventricular pressures and myocardial stretch^26^. The role of natriuretic peptides in AHF has been extensively evaluated in patients with HFrEF, and the evidence is scarcer for HFpEF^27,28^. Most prior studies in HFpEF evaluated mortality or the composite of death and readmission, with a prognostic value not different from those with HFrEF^27,28^. The reasons behind the lack of predictive ability of NT-proBNP for predicting total AHF readmission in this sample remain elusive. However, some reasons have been postulated.

1. The higher prevalence of right-sided dysfunction and systemic congestion in HFpEF over HFrEF has been previously reported^29^. Thus, the relevance of CA125 can be expected to be a surrogate of systemic congestion over NT-proBNP as a proxy of left-sided filling pressure for predicting morbidity burden in this population. Thus, patients with HFpEF and predominant right-sided HF CA125 but not NTproBNP has been associated with worse outcomes^30^.
2. Patients with HFpEF are frequently elderly and display a higher prevalence of renal dysfunction, situations in which natriuretic peptides are elevated regardless of the severity of HF^26^. Indeed, a recent study from our group showed that the main factors associated with NT-proBNP in HF patients were renal dysfunction, LVEF, and age^25^. Conversely, clinical parameters of congestion and the severity of tricuspid regurgitation were the most important predictors for CA125^25^.
3. Additionally, there is compelling evidence that natriuretic peptides are not accurate or reliable markers of tissue congestion^26^.
4. NT-proBNP was measured early during hospitalization. Prior studies have suggested predischarge natriuretic peptides assessment might have greater prognostic ability^31^.

Beyond the pathophysiology supporting the positive association between CA125 and burden of total HF-admission, we envision that the assessment of CA125 during decompensation might be a helpful complementary tool for predicting the risk of subsequent new HF decompensations. Thus, circulating levels of CA125 may play a role in planning the intensity of depletion therapy^32,33^, length of stay^34^, and frequency of postdischarge monitoring as reported in recent studies in which high CA125 identified patients that benefit from more intensive diuretic regimens, longer hospital stays and close postdischarge follow-up^32–34^. The current study expands the relevant role of CA125 as a circulating biomarker in patients with HF by confirming its value for predicting the morbidity burden in a frequent syndrome in elderly and comorbid patients in which most of the available therapeutic strategies remain empirical.

Some limitations need to be acknowledged. First, this study has the inherent limitations of being an observational study in which HF admission policies/criteria may differ from other healthcare systems. Additionally, despite a robust multivariate adjustment, other confounders may be involved. Furthermore, our conclusions cannot be extrapolated to patients with stable chronic HFpEF. Third, information regarding LVEF values prior to admission was not recorded, so a possible differential risk prediction in those with recovered ejection fraction was not evaluated. Fourth, we cannot evaluate the prognostic role of serial CA125 and NT-proBNP measurement with the current design. Lastly, both bioamarkers were measured at early hospitalzations. It may have not relevant consequences for CA125 because it long-half life (7–12 days)^5^. However, it might represent a limitation for NTproBNP in which predischarge assessment has shown a prognostic superiority over early hospital assessment^31^.

In conclusion, in patients with HFpEF discharged after an episode of AHF, CA125 predicted the risk of the total burden of AHF-readmission. Further studies should confirm these findings.

## Supplementary Information

Supplementary Information 1.

Supplementary Information 2.

Supplementary Information 3.

Supplementary Information 4.
