# Supplementary figures and images for: Carbohydrate antigen 125 and risk of heart failure readmissions in patients with heart failure and preserved ejection fraction
Source: Sci Rep. 2022 Jan 25;12:1344. doi: 10.1038/s41598-022-05328-2 (PMC8789924; doi:10.1038/s41598-022-05328-2)

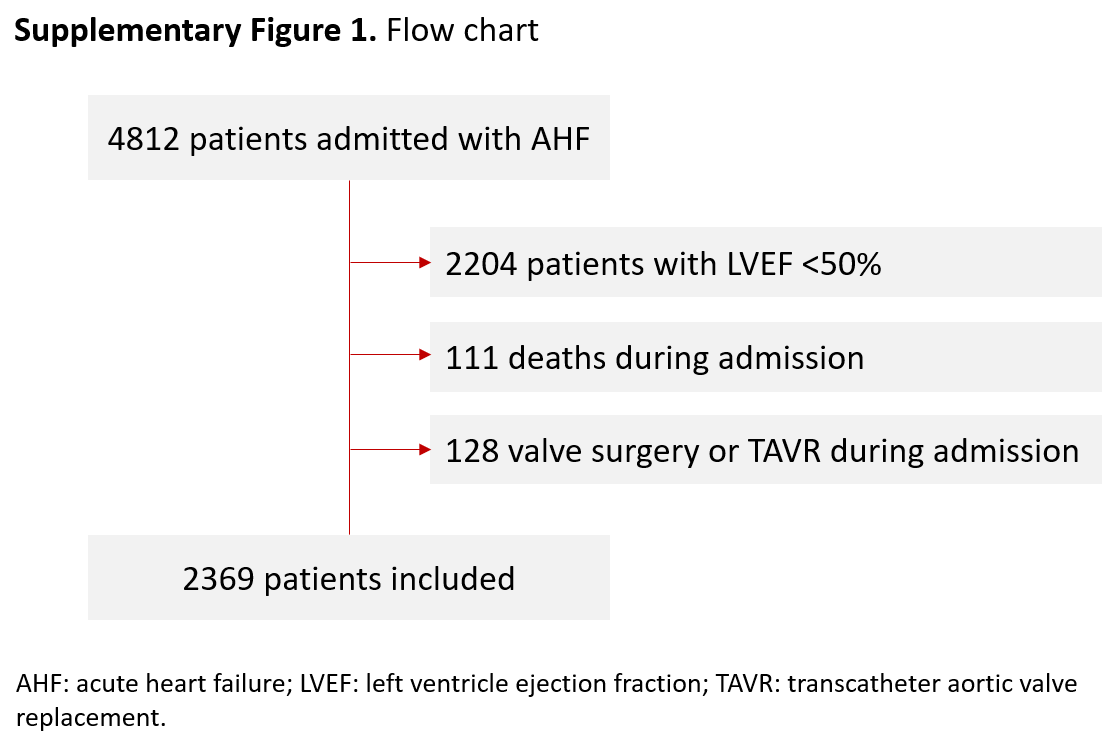

Supplement: Supplementary file 1 — Supplementary Information 1. [file 41598_2022_5328_MOESM1_ESM.tif]

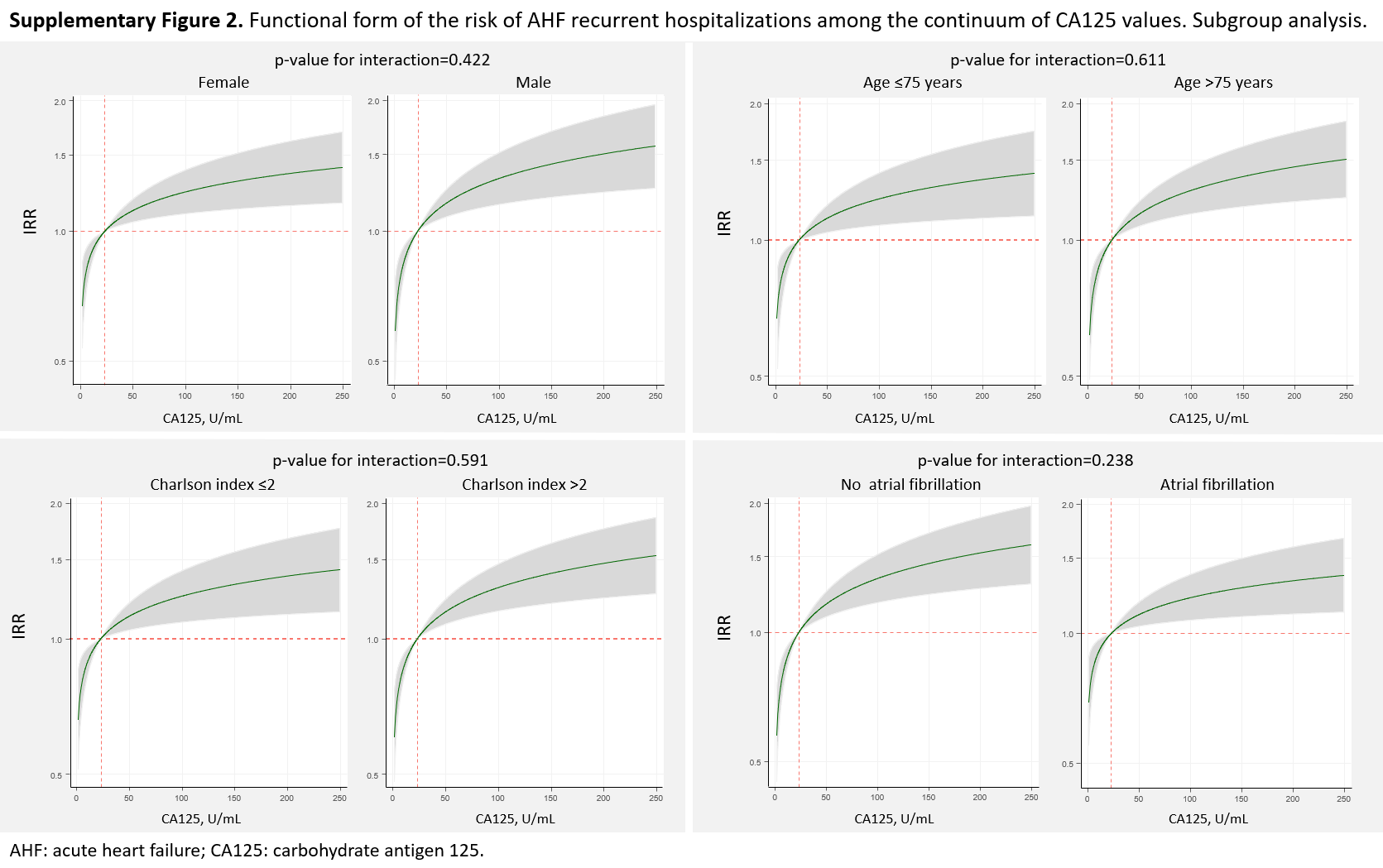

Supplement: Supplementary file 2 — Supplementary Information 2. [file 41598_2022_5328_MOESM2_ESM.tif]

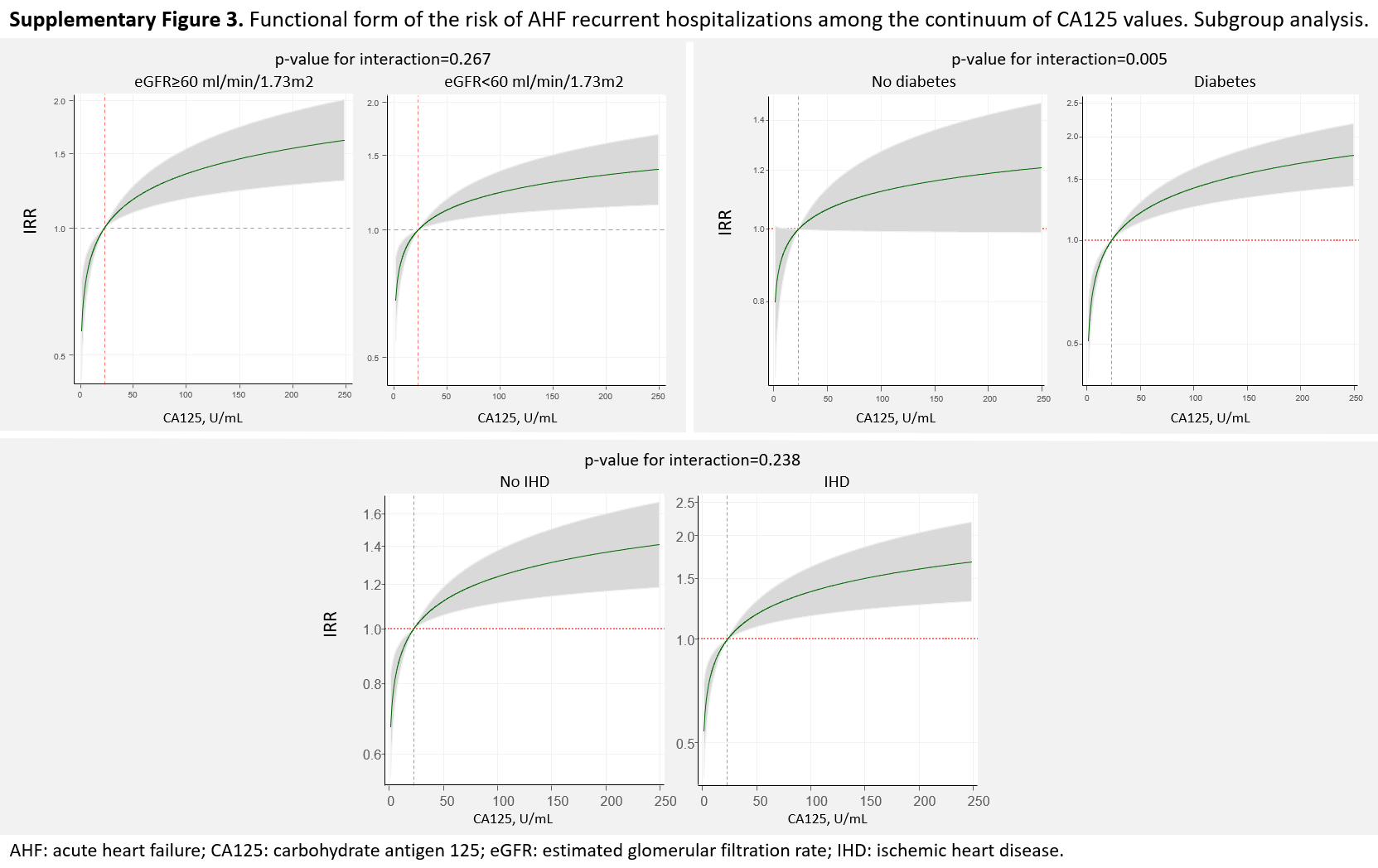

Supplement: Supplementary file 3 — Supplementary Information 3. [file 41598_2022_5328_MOESM3_ESM.tif]

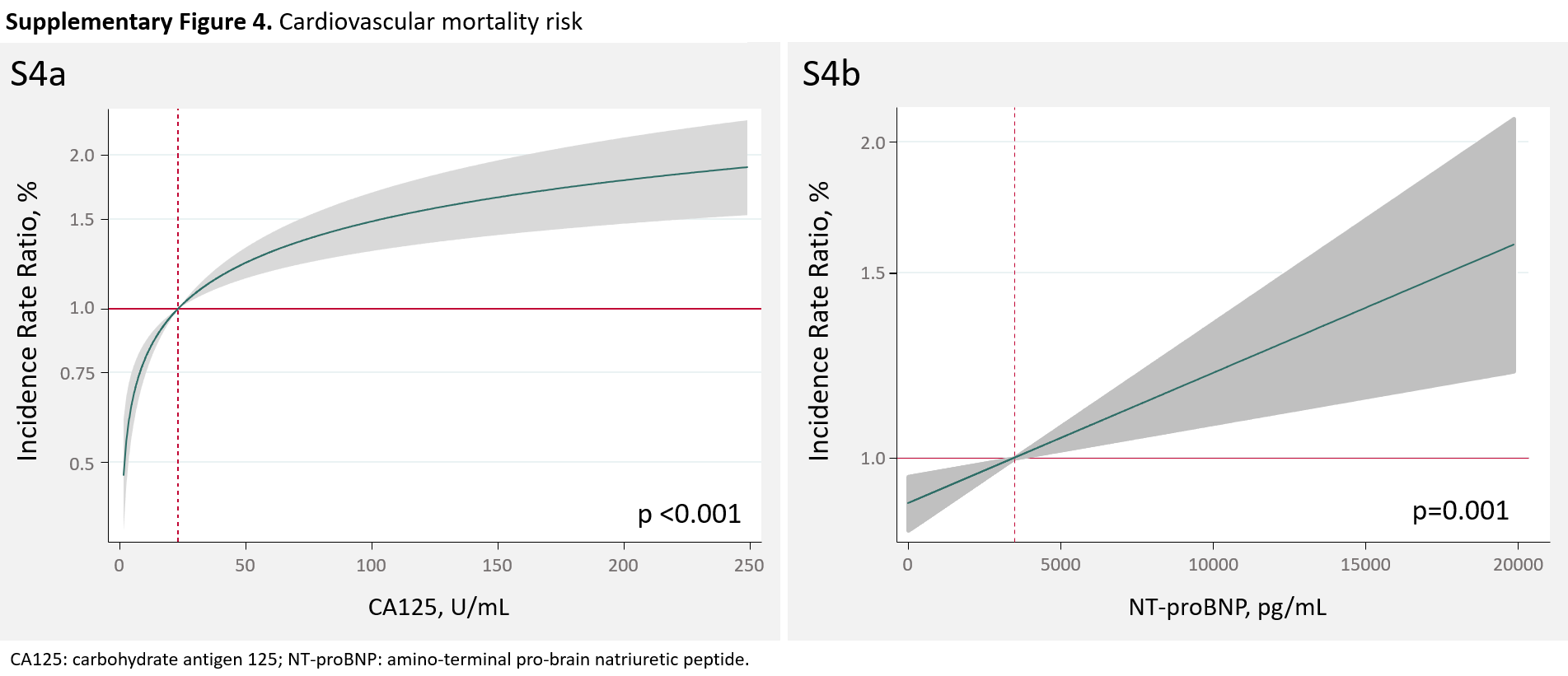

Supplement: Supplementary file 4 — Supplementary Information 4. [file 41598_2022_5328_MOESM4_ESM.tif]
